# Static versus dynamic intensity-modulated radiotherapy: Profile of integral dose in carcinoma of the nasopharynx

**DOI:** 10.4103/0971-6203.51932

**Published:** 2009

**Authors:** K. S. Jothybasu, Amit Bahl, V. Subramani, G. K. Rath, D. N. Sharma, P. K. Julka

**Affiliations:** Department of Radiation Oncology, All India Institute of Medical Sciences, New Delhi-110 029, India

**Keywords:** Dynamic multileaf collimator, integral dose, intensity-modulated radiotherapy, static multileaf collimator

## Abstract

This study is aimed to evaluate the impact of static and dynamic intensity modulated radiotherapy (IMRT) delivery techniques planned with Eclipse TPS on the integral dose to the healthy normal tissue surrounding the tumor-bearing area and to the volume receiving doses < 5 Gy in patients with carcinoma nasopharynx treated with Simultaneous Integrated Boost IMRT (SIB-IMRT). Ten patients with carcinoma nasopharynx were chosen for this dosimetric study. IMRT plans were generated with 6X using dynamic multileaf collimator (DMLC) and static multileaf collimator (SMLC) with 5, 10 and 15 intensity levels (L). Integral dose, volume receiving 5 Gy, number of monitor units (MU) is compared against DMLC. The mean difference in the MU delivered per fraction between 5, 10 and 15 L SMLC and DMLC was −13.25% (*P* < 0.001, with paired t test), −11.82% (*P* < 0.001) and −10.81% (*P* < 0.001), respectively. The mean difference in the integral dose with 5, 10 and 15 L compared to DMLC was −2.96% (*P* < 0.001), −2.67% (*P* = 0.016) and −0.39% (*P* = 0.430), respectively. However, the difference in low-dose volume (V5Gy) was statistically insignificant with mean difference of 0.60% (*P* = 0.23), 1.18% (*P* = 0.017) and 1.70% (*P* = 0.078), respectively for 5, 10 and 15 L compared to DMLC. Our results show that while choosing the IMRT delivery technique using conventional MLC the concerns about integral dose and volume receiving very low doses such as 5 Gy can be ignored.

## Introduction

Intensity-modulated radiotherapy (IMRT) has become an important radiation delivery technique in the management of head and neck cancer. IMRT offers several advantages over three-dimensional conformal radiotherapy (3DCRT). IMRT conform the prescribed doses to the target volumes of complex shapes while sparing the adjacent critical structures without compromising the target coverage. Moreover, IMRT can enhance the fluence at the margins of the target and compensate for the beam penumbra without extending the portal boundaries. Another distinct advantage offered by IMRT is that it makes it possible to deliver different doses to different target volumes in a single plan, commonly referred to as Simultaneous Integrated Boost IMRT (SIB-IMRT). Mohan *et al.* have shown that IMRT distributions are most conformal when designed to be delivered as SIB-IMRT.[1] Normal tissues outside the treated volumes are at reduced complication risk in such techniques as they receive a lower total dose as well as lower dose per fraction.

IMRT can be delivered using a conventional multileaf collimator (MLC), binary MLC or using a physical compensator. Among the three, conventional MLC is the most commonly used. IMRT delivery using a conventional MLC involves either a segmental MLC (SMLC)-based or dynamic MLC (DMLC)-based approach. Although the former involves delivery of radiation when MLC leaves are stationary, in the latter case MLC leaves are moving as the radiation is delivered. The main advantage of using a DMLC is that the continuous leaf motion enables the delivered intensity to closely match with the optimal fluence calculated by the inverse treatment planning algorithm (ITP), accurately preserving both the spatial and intensity resolutions. On the other hand, an SMLC approach resembles a conventional multi-segmented treatment and requires approximating the intensity profile into discrete intensity levels (briefly described in the methods and materials section), resulting in a lower resolution.[2] The SMLC IMRT may be convenient to verify and is technically less demanding than a DMLC treatment. A DMLC-based delivery requires more monitor units (MU) than an SMLC method, as the beam is kept on throughout the delivery of radiation.[3] The leakage radiation from collimator leaves and scattered radiation is also different for the two delivery techniques. A difference in integral dose delivered to the surrounding tissues or the volume receiving low dose is thus expected between the two methods due to the difference in the required MU to deliver the same prescription dose. Kry *et al.* have shown that depending on the treatment energy, IMRT using step and shoot requires 3.5–4.9 times more monitor units as compared to the conventional treatment.[4] These figures are likely to increase with the use of dynamic IMRT. Chui *et al.* have shown that a dynamic IMRT requires 20% more MU as compared to static IMRT.[5] Alaei *et al.* have shown that SMLC on an average required 15% lesser MUs than a DMLC with 15% longer treatment time than an SMLC treatment.[6] This can lead to an increase in the low-dose volume as well as the risk of radiation-induced malignancies. The issue of integral dose or the total cumulative dose received by tissues is clinically relevant because of the anticipated higher risk of second malignancies associated with a higher integral dose.[7,8]

The choice of delivery technique, static or dynamic, has become a topic of debate due to substantial difference between the MUs required to deliver the same treatment. Increased MU is expected to increase the integral dose and the low-dose volume. As indicated by Hermanto *et al.* few studies have addressed the effect of IMRT on the volumes receiving very low doses, such as 5 Gy, which may be more relevant to increasing the risk of second malignancies.[9] At higher doses the risk of inducing cancer will decrease due to dominant cell killing rather than cell mutations.[10] The differences between the MUs required to deliver the same treatment is attributed to the method of delivery. There are few studies that compared SMLC and DMLC in terms of dosimetric quality of the plans.[5,6] Other studies have analyzed the impact of number of beams, beam energy and the delivery technique on integral dose.[11,12] These techniques have shown that integral dose has < 1% variation with number of beams and higher beam energies reduced the integral dose. No studies have compared DMLC and SMLC techniques with regard to integral dose and low-dose volume, which have a significant effect on the probability of radiation-induced malignancies. The main objective of this study is to evaluate the effect of the two IMRT delivery techniques DMLC and SMLC, planned using Eclipse (Varian Associates, Palo Alto, CA), on the integral dose to the healthy normal tissue surrounding the tumor-bearing area and also the volume receiving doses < 5 Gy. We have chosen nasopharynx cases as the complex shape and spatial location of the target and critical structures demand a more complex fluence profile than many other sites.

## Materials and Methods

Ten cases of carcinoma of nasopharynx treated by SIB-IMRT were chosen for this study. Planning computed tomography (CT) images with a slice thickness of 2.5 mm were obtained for all patients while immobilized in treatment position. Target volumes and Organs at risk (OAR) were contoured using Eclipse TPS (Varian Associates, Palo Alto, CA). The gross tumor volume (GTV) included the radiologically apparent primary tumor and involved lymph nodes. CTV1 (clinical target volume) included the GTV and adjacent soft tissue and nodal regions. The CTV2 included the elective nodal regions. A dose of 70 Gy to GTV, 59.4 Gy to CTV1 and 54 Gy to the CTV2 were prescribed in 33 fractions. A 5-mm margin was given to the CTV to the planning target volume (PTV). For each patient IMRT plans were generated using Eclipse's Dose Volume Optimizer using default fluence smoothing parameters (40 in X and 30 in Y) with a Varian Clinac 2300CD linear accelerator, which is capable of delivering both Static and Dynamic IMRT with 40 leaf-pair MLC, which is built-in the accelerator as a tertiary collimator. Nine equispaced coplanar beams were used to generate the IMRT treatment plans. The dose-volume constraints used in treatment planning are shown in Table 1. All plans were made with 6 MV photons and doses were calculated for DMLC delivery technique with a grid size of 2.5 mm with Pencil beam convolution algorithm. All plans were normalized at the isocenter and the prescription isodose surface was chosen such that at least 95% of the target volume receives the prescription dose. Dummy structures were also drawn wherever necessary to avoid dose spillage in the normal tissues. Later on, all the plans were converted to Static IMRT using the Leaf Motion Calculator (LMC) with 5, 10 and 15 intensity levels (L). A short description of the Varian DMLC and SMLC delivery method is given in the subsequent sections.

**Table 1:** Dose-Volume constraints used in the planning

*Structure*	*Clinical dose limits (Gy)*	*Inverse planning constraints*	
		
		*Volume (%)*	*Dose limit (Gy)*
GTV-70	D98 ≥70	100	≥66.5 (5% < 70)
		1	≤77 (10% > 70)
PTV-59.4	D95≥59.4	100	≥57% (5%< 59.4)
		1	≤66 (10% >60)
PTV-54	D95≥54	100	≥51.5 (5% <54)
		1	≤59.5 (10% >54)
Brainstem	Dmax≤54	0	≤54
Spinal cord	Dmax≤45	0	≤ 45
Parotid	Dmean≤26		
Retina	Dmax≤45	0	≤45

**Figure 1:**
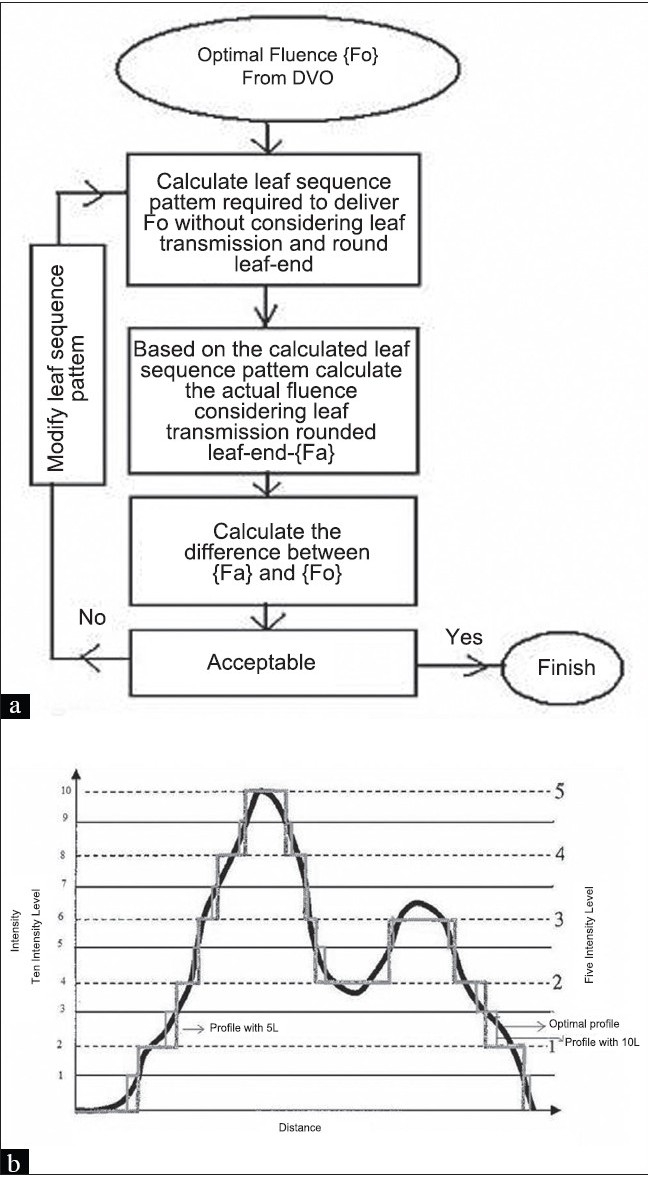
(a) Flow chart of LMC, (b) Discretization of fluence profiles with different intensity levels in SMLC

The spatial resolution of the intensity distribution used in this study is 2.5 mm in the leaf travel direction and 1 cm in the direction perpendicular to the leaf travel, which is limited by the physical thickness of the leaf. The dose rate used for the delivery was 300 MU/min and the maximum leaf speed was set to 2.5 cm/s. The basic idea behind the sliding window method is to sweep the MLC leaf pairs of varying aperture sizes and speed over a field. The main advantage of this technique is that by properly choosing the speed and aperture sizes, it is possible to deliver any intensity profile. After the calculation of the optimal fluence by the Dose Volume Optimizer (DVO) that is necessary to achieve the desired dose distribution in the patient, the fluence profile is transferred to the LMC program to calculate the necessary leaf sequence pattern. While calculating the leaf sequence pattern the LMC takes into account the physical limitations of the MLC device (eg, minimum leaf gap that should be maintained to avoid collisions between leaves, maximum leaf travel, etc.), transmission and leakage through the leaves (ie, the rounded leaf-end transmission). While calculating the leaf sequence file, the LMC program optimizes the leaf sequence pattern iteratively to reduce the MU required to deliver the optimal fluence profile and also to keep the delivered and the optimal fluence profiles as close as possible. However, due to the inherent limitations of the delivery device, it is impossible to deliver exactly the same fluence profile as calculated by the DVO. Due to this reason, the delivered and the optimal fluence profiles are never the same. The final fluence or the fluence to be delivered is calculated by the LMC based on the final leaf sequence pattern. In order to predict the delivered dose distribution as accurately as possible, the dose calculation algorithm uses the actual fluence calculated by the LMC for the final dose calculation. For fields wider than the Effective Leaf Out of Carriage Distance (ELOC) (in other words, if the leaves are not able to reach the opposite edge of the field) the original field is split into subfields, which are narrower than the ELOC and a sliding window leaf motion plan is calculated for each subfield separately. Because it is practically impossible to accurately connect sharp edges of subfields, the subfields are given an overlapping area. When the beam is turned off for a carriage shift, all leaf pairs are required to form a window inside the overlapping area. An optimization strategy is implemented in the LMC program to determine the number of subfields required and where to split the fields. The flow chart of the LMC algorithm is given in Figure 1a.

In the SMLC method, the continuous fluence profile is divided into equally spaced discrete intensity levels, as shown in Figure 1b. Even though it is possible to divide a continuous profile into unequally spaced intensity levels, such strategies are less commonly used. The LMC in Eclipse treatment planning system equally divides the continuous fluence profile with a user-defined input for individual fields. The spatial resolution used in this study for SMLC is the same as that for DMLC. The framework used for leaf sequencing is almost the same for both DMLC and SMLC. The sliding Window algorithm is first applied to the optimal fluence calculated by the DVO and the leaf trajectories are sampled to a smaller number of segments. The number of segments to be sampled depends on the intensity level defined for that field and the maximum fluence value calculated for that field. For example, with 5 intensity levels and a maximum fluence (transmission factor) value of 2, we will have 10 sub-fields for that particular field. After this process, the sub-fields are fine-tuned by incorporating transmission and leakage through the leaves similar to DMLC. The greatest disadvantage of this method is that the continuous fluence profile is rounded to the nearest discrete intensity level. An example profile is given in Figure 1b, where it shows the approximation of a continuous profile in to 5 and 10 intensity levels. It is evident from Figure 1b that as the intensity level increases, the delivered profile becomes closer to the optimal profile. However, increasing the intensity level will also increase the number of sub-fields. For example, an IMRT field with 5-intensity level with a maximum transmission factor of 2 will have 10 sub-fields, and a field with the same maximum transmission factor and an intensity level of 20 will have 40 sub-fields.

The Integral Dose (ID) was calculated as the mean dose times the volume of the structure as given in equation (1). To calculate the ID to the normal tissues outside the target volumes, all the target volumes were subtracted from the body volume (body minus target volumes) and referred to as the normal healthy tissue (NHT). The volume receiving 5 Gy (V5) or less than what was determined from the dose-volume histograms (DVH) calculated for the NHT. All the plans were later converted to deliver by the SMLC method, using the same optimal fluence generated for the sliding window technique with intensity levels of 5, 10 and 15. We calculated the volume integral over mass integral, as the mass integral would misrepresent the contribution of a structure with highly heterogeneous density to non-target integral dose as it is the case in head and neck.[11] The number of MU required for all the techniques were also investigated.

(1) Integral Dose = Mean Dose × Volume (Liter-Gray)

The dose to target volumes and critical structures were also compared for different techniques. Statistical analyses were performed using a paired two-tailed Student *t* test to determine whether there is any statistically significant difference in any of the parameters examined. Differences were considered statistically significant with *P* ≤ 0.05.

## Results

**Figure 2:**
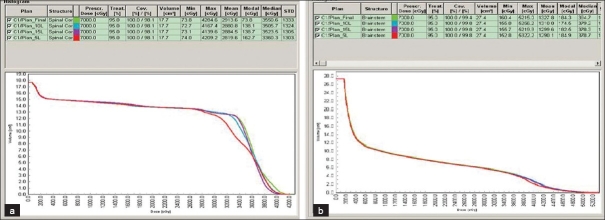
(a) DVH for spinal cord in DMLC and SMLC, (b) DVH for brainstem in DMLC and SMLC

**Figure 3:**
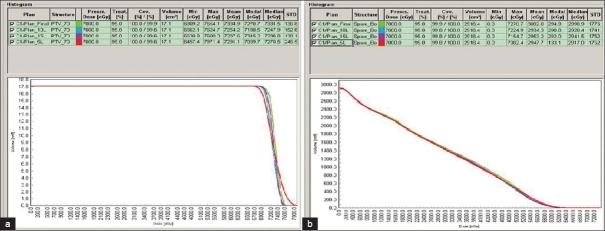
(a) DVH for PTV in DMLC and SMLC, (b) DVH for Normal healthy tissue in DMLC and SMLC

**Figure 4:**
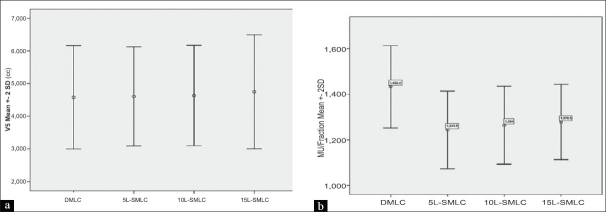
(a) Error bars of V5 for different delivery techniques, (b) Error bars of MU for different delivery techniques

**Figure 5:**
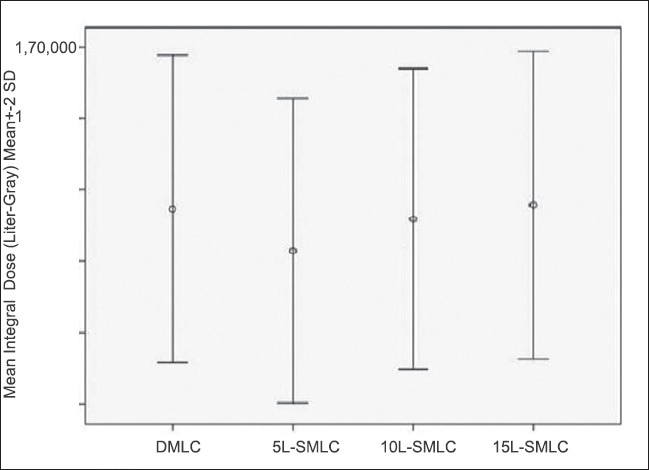
Error bars of mean integral dose for different delivery techniques for NHT

The dosimetric results were almost similar for the DMLC and SMLC plans with 10 and 15 L. The SMLC plan with 5 L slightly deviated from the prescribed dose-volume constraints. Figures 2 and 3 present DVH for a typical patient plan with regard to PTV, brainstem, spinal cord, parotid and NHT along with the dose statistics. The mean difference in the MU delivered per fraction between 5 L SMLC and DMLC delivery was −13.25% (*P* < 0.001, with paired *t* test).The difference with other intensity levels varied very less compared to 5 L, with a difference of −11.82% (*P* < 0.001) for 10 L and −10.81% (*P* < 0.001) for 15 L. The difference between the SMLC delivery with 10 and 15 L compared with 5 L was 1.65% (*P* < 0.001) and 2.81% (*P* < 0.001), which may not increase the transmission and leakage dose significantly. However, 5 L SMLC delivery slightly degraded the PTV dose uniformity (DVH for a sample patient is given in Figure 3a) while reducing the high dose volume in the spinal cord. All other critical structure doses were within the tolerance limits for all the delivery techniques. The number of MU required to delivering the same dose varied between DMLC and SMLC, but the difference between SMLC with different intensity levels varied less. The mean difference between the DMLC and 5 L SMLC was −13.25% (*P* < 0.001). The mean difference in the integral dose observed with 5, 10 and 15 L compared to DMLC was −2.96% (*P* < 0.001), −2.67% (*P* = 0.016) and −0.39% (*P* = 0.430), respectively. However, the difference in the low dose volume (V5 Gy) was statistically insignificant with a mean difference of 0.60% (*P* = 0.23), 1.18% (*P* = 0.017) and 1.70% (*P* = 0.078), respectively for 5, 10 and 15 L compared to DMLC; Table 2). The error bars are given in Figures 4 and 5 for V5, integral dose and monitoring units.

**Table 2:** Mean difference of MU, Integral dose and V5 between dynamic multileaf collimator and static multileaf collimator delivery techniques

*Delivery technique*	*DMLC Mean*	*5 L*			*10 L*			*15 L*		
				
		*Mean*	*% Diff*	*p*	*Mean*	*% Diff*	*p*	*Mean*	*% Diff*	*p*
MU Integral	1433.40 ± 90.13	1243.5 ± 85.20	−13.25	<0.001	1264 ± 85.38	−11.82	<0.001	1278.50 ± 82.59	−10.81	<0.001
Dose (Liter-Gray)	143.58 ± 28.6	139.25 ± 27.39	−2.96	<0.001	139.87 ± 28.74	−2.67	0.016	143.11 ± 28.97	−0.39	0.430
V5 Gy (cc)	4578.21 ± 792.37	4605.67 ± 760.19	0.60	0.23	4632.12 ± 770.7	1.18	0.017	4748.37 ± 873.39	1.70	0.078

## Discussion

**Figure 6:**
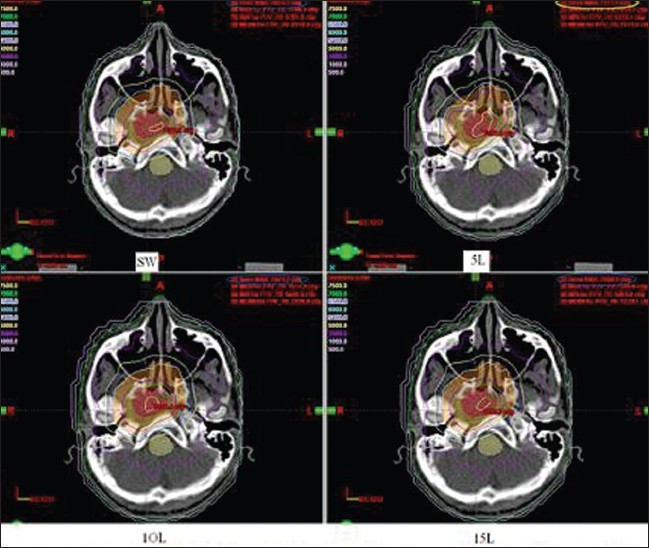
Dose distribution for different delivery techniques

**Figure 7:**
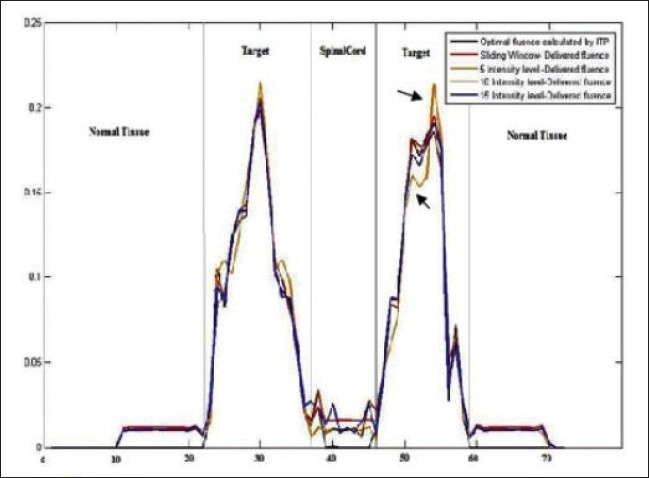
Deliverable fluence profiles superimposed on optimal fluence profiles

Integral dose or the total cumulative dose to normal untreated tissues is higher in IMRT as compared to conventional treatment.[13,14] In this study, we have compared the integral dose and the low-dose volume in the normal healthy tissues with SMLC and DMLC. Although DMLC increased the integral dose to NHT, no significant difference was found in the volume receiving 5 Gy when compared with SMLC with 5, 10 and 15 L. SMLC with low intensity levels such as 5 L slightly degraded the dose uniformity in the target volumes. For example, in Figure 6, which shows the axial dose distribution with DMLC, SMLC with 5, 10 and 15 L the global dose maximum is higher in 5 L plan (highlighted with yellow ellipse), whereas in other plans it is almost the same (DVH of PTV in Figure 3a). SMLC decreased the high-dose volume in the spinal cord while maintaining the dose maximum within tolerance limit. The high-dose volume in the spinal cord with 5 L SMLC delivery is less than what is used in all other methods. This can be attributed to the less number of MU required and also due to change of field shapes in SMLC when the beam is kept off. In DMLC the beam is continuously switched on, which increases the dose to the OARs due to transmission and leakage through the leaves. Moreover, a DMLC delivery cannot completely shield any area rather it sweeps the area with minimal gap at a maximum speed possible. The dose uniformity degradation in the target volumes can be explained by comparing the delivered fluence patterns of different plans. Figure 7 shows comparison of delivered fluence profiles with optimal fluence profile calculated by the DVO taken along the center of the field across the patient body. It is evident that 5 L SMLC delivery was unable to reproduce the optimal fluence profile calculated by the DVO. In the regions where the optimal fluence profile changes abruptly, 5 L SMLC delivery differed significantly from the optimal fluence profile with overdose and underdose, which reflects in the target dose uniformity (arrow marks in Figure 7). However, no significant difference was seen in the profiles with 5 L SMLC in OAR and normal tissue regions, which results in more or less same OAR doses.

The integral dose in the NHT did not vary much with different intensity levels in SMLC delivery, as there is no significant difference in the number of MU delivered. Our statistical analyses showed that a significant difference exists between DMLC and 5 and 10 L SMLC but not between DMLC and 15 L SMLC. The low-dose volume, which is considered a potential volume to develop radiation-induced second malignancies, did not differ much. The role of neutrons in the integral dose and low-dose volume is ruled out in low energies as the threshold for neutron production is about 10 MeV.

There are proponents of both SMLC and DMLC techniques of IMRT delivery. Using a large number of intensity levels can make a SMLC method almost equivalent to DMLC. However, treatment time may be prohibitively long with such approach. Other advantages of SMLC include a simpler MLC control system and fewer monitoring units compared to dynamic treatments. Quality assurance for a SMLC plan is also easier than in DMLC. Nevertheless, a DMLC delivery can be faster than a SMLC delivery as the beam is continuously on.[5,15] Studies have shown that a leaf position error of 1 mm can result in 10% error in dose delivery, which demands extremely regular and stringent quality assurance for the entire delivery system.[16]

This study is an attempt to-quantify the difference in the integral dose and low-dose volume with static and dynamic IMRT planned using Eclipse TPS delivered by a conventional linac with conventional MLC. The accuracy of the results in this study is highly dependent on the accuracy of the dose calculation models used in this study. The dose calculation models used in this study do not account for MLC scatter, collimator leakage and tongue-and-groove effect. The incorporation of these effects in the dose calculation models is likely to increase the difference in the results. Recently, Jang *et al.* have compared conventional dose calculation models with Monte Carlo methods, and have shown that conventional dose calculation models do not properly model the secondary radiation from MLC, which contributes significantly to the low-dose in the IMRT plans.[17] A more accurate dose calculation algorithm such as Monte Carlo method will provide more accurate results in such studies.

## Conclusions

DMLC-based IMRT slightly increased the integral dose to normal healthy tissues when compared to SMLC-based delivery. However, no significant difference was found in the low-dose volume with all the techniques despite having a significant difference in the number of MU required. We conclude that while choosing the IMRT delivery technique using conventional MLC the concerns about integral dose and the volume receiving very low doses such as 5 Gy can be ignored while using Eclipse TPS.
